# Effects of chronic light cycle disruption during adolescence on circadian clock, neuronal activity rhythms, and behavior in mice

**DOI:** 10.3389/fnins.2024.1418694

**Published:** 2024-06-17

**Authors:** Pablo Bonilla, Alexandria Shanks, Yatin Nerella, Alessandra Porcu

**Affiliations:** Department of Drug Discovery and Biomedical Science, University of South Carolina, Columbia, SC, United States

**Keywords:** light cycle disruption, suprachiasmatic nucleus, dentate gyrus, medial amygdala, somatostatin, clock genes, neuronal activity, avoidance behavior

## Abstract

The advent of artificial lighting, particularly during the evening and night, has significantly altered the predictable daily light and dark cycles in recent times. Altered light environments disrupt the biological clock and negatively impact mood and cognition. Although adolescents commonly experience chronic changes in light/dark cycles, our understanding of how the adolescents’ brain adapts to altered light environments remains limited. Here, we investigated the impact of chronic light cycle disruption (LCD) during adolescence, exposing adolescent mice to 19 h of light and 5 h of darkness for 5 days and 12 L:12D for 2 days per week (LCD group) for 4 weeks. We showed that LCD exposure did not affect circadian locomotor activity but impaired memory and increased avoidance response in adolescent mice. Clock gene expression and neuronal activity rhythms analysis revealed that LCD disrupted local molecular clock and neuronal activity in the dentate gyrus (DG) and in the medial amygdala (MeA) but not in the circadian pacemaker (SCN). In addition, we characterized the photoresponsiveness of the MeA and showed that somatostatin neurons are affected by acute and chronic aberrant light exposure during adolescence. Our research provides new evidence highlighting the potential consequences of altered light environments during pubertal development on neuronal physiology and behaviors.

## Introduction

Light is crucial for most species, including humans, to survive and thrive. Besides its role in the generation of vision, light modulates a wide variety of physiological functions such as the biological rhythm, sleep, arousal, and mood (Tancredi et al., 2022; Zielinska-Dabkowska et al., 2023). Historically, human life was synchronized with predictable daily light and dark cycles driven by the solar day, aligning physiology and behaviors with the natural environmental rhythm. However, with the widespread adoption of electric light, individuals are increasingly subjected to altered light environments characterized by various light sources that disrupt the natural day-night cycle (Bedrosian and Nelson, 2017). Currently, more than 80% of the world and more than 99% of the US and European populations live under light-polluted skies (Falchi et al., 2016). Indoor light exposure has increased during the last decades, mainly because of home lights turned on during the night, as well as new sources of exposure (e.g., electronic devices such as monitors, smartphones, etc.), polluting the natural nighttime darkness and exerting potential risks to human health (Smolensky et al., 2015; Lunn et al., 2017). Exposure to altered light environments has been linked to detrimental effects in humans, including increased risk of breast cancer, obesity (Lai et al., 2020; Luo et al., 2023), and psychiatric disorders (Bedrosian and Nelson, 2017; Tancredi et al., 2022). Of note, recent studies have linked altered light environments to increased anxiety and mood disorders among US adolescents (Paksarian et al., 2020). Adolescents, with their widespread use of electronic devices coupled with altered sleep/wake habits, experience greater exposure to chronic light cycle disruption than any previous generation (Hysing et al., 2015). However, the effects of altered light environment on brain circuits and behaviors during pubertal development remain understudied.

Direct effects of light on circadian rhythms were first emphasized by Aschoff (1999), introducing the term “masking” to describe them. Initially, efforts focused on minimizing masking effects to accurately measure circadian phase (Minors et al., 1996; Mrosovsky, 1999). However, this led to a negative view of masking (Rensing, 1989). From a broader perspective, masking may complement circadian control by facilitating appropriate timing of behavior and physiology, correcting circadian clock synchronization errors, and refining activity patterns (Redlin, 2001; Thompson et al., 2008; Rotics et al., 2011). Pioneer studies highlighted how the combined output of endogenous oscillators and direct light response has been optimized through evolution (Rensing, 1989; Gonze, 2000; Roenneberg and Merrow, 2002). Despite these implications, our understanding of the mechanisms behind light’s direct influence on daily patterns and its significance for temporal organization in nature remains limited.

In mammals, intrinsically photosensitive retinal ganglion cells (ipRGCs) expressing melanopsin are responsible for non-image-forming light detection (Hattar et al., 2006; LeGates et al., 2012). IpRGCs were thought to be a uniform neuronal population whose predominant role was to influence circadian rhythms, as they project to the suprachiasmatic nucleus (SCN), the central circadian pacemaker (Gooley et al., 2001; Berson et al., 2002). Recently, Fernandez et al. showed that a subset of ipRGCs that project to the SCN, are sufficient to drive light-mediated cognitive deficits without disrupting the SCN clockwork machinery. At the same time, an SCN-independent pathway mediates light-induced mood changes (Fernandez et al., 2018). Indeed, ipRGCs show widespread projection patterns throughout many other regions of the rodent brain (Hattar et al., 2006; Li and Schmidt, 2018), including the medial amygdala (MeA) (LeGates et al., 2014), a key region regulating innate avoidance/approach behaviors (Hong et al., 2014; Miller et al., 2019). IpRGCs are also most sensitive to blue wavelengths of light, with a peak sensitivity of ~482 nm (Berson et al., 2002; Lockley and Gooley, 2006). A number of studies and models have been used to investigate the effects of altered light environment on circadian rhythms, mood, learning, and memory in adult rodents, including constant light (Ma et al., 2007), social jet lag (Delorme et al., 2022), T7 cycle (3.5 h light and 3.5 h dark) (LeGates et al., 2012), dim light at night (Fonken et al., 2009, 2012; Bedrosian et al., 2011) and phase shifts (Gibson et al., 2010; Loh et al., 2010). In contrast to adults, little is known about the effects of altered light environment exposure during adolescence development. Animal studies revealed that mice raised under aberrant lighting conditions develop avoidance behaviors in adulthood (Borniger et al., 2014; Cissé et al., 2016), suggesting an important role of the early life lighting environment on affective development. Despite being a critical neuronal circuit maturation period, adolescence has historically been overlooked but is now the subject of intensive investigation due to its unique sensitivity to environmental stimuli (Tooley et al., 2021). Indeed, identifying the effect of altered light environment on brain circuits during pubertal development will provide new mechanistic data that might be useful for understanding the increased affective disorders in adolescents in recent years (Twenge et al., 2019), and whether these detrimental effects can be prevented or reversed.

To start addressing the effects of altered light environment on brain circuits and behaviors during adolescence, we developed a light protocol for chronic light cycle disruption and implemented it in adolescent mice. Human adolescent light/dark exposure is heavily influenced by late sleep onset, combined with early wake-up time on school days and late wake-up time on weekends (Hasler et al., 2012; Gamble et al., 2014; Mireku et al., 2019; Vetter et al., 2019). The early wake-up time on weekdays combined with the wide use of bright electronic devices at night leads to an extended light phase for 5 days a week among adolescents. To mimic this chronic light cycle disruption (LCD) we exposed adolescent mice to 19 h of light and 5 h of darkness (19 L:5D) for 5 days and 12 L:12D for 2 days, for a total of 4 weeks. In this model, LED bright light is turned on during the night phase of the animals’ cycle, resulting in increased duration of light exposure for 5 days of a 7-day cycle, for a total of 4 weeks. We explored the effects of this new light paradigm on circadian locomotor activity, memory, and avoidance behaviors. We also explored circadian clock gene expression and neuronal activity rhythms in the SCN, hippocampus, and MeA. Finally, we unveiled the MeA neuronal subpopulations responsive to light. Our data indicate that chronic light cycle disruption during adolescence decreased memory and increased avoidance behavior associated with changes in circadian rhythms and neuronal activity without affecting the circadian pacemaker.

## Materials and methods

### Mice

Female and male mice used in this study were on a C57BL/6 J genetic background, except for GAD67- GFP mice that were on CD1 background. The Gad1-tm1.1Tama (GAD67-GFP knock-in) mouse line was provided by Y. Yanagawa (Gunma University Graduate School of Medicine, Japan). Mice were heterozygous for insertion of the gene encoding GFP under the control of the GAD67 gene promoter. They were used to label the inhibitory GABAergic neurons by enhancing the GFP signal with an anti-GFP antibody. Mice were bred and reared in an on-site animal facility at UofSC under controlled environmental conditions and maintained in standard group housing cages with *ad libitum* access to normal chow and water. Light intensity was approximately 600 lux at the top of the cage, and the ambient temperature was 23° ± 2°C. The time referred to as “lights on” is defined as ZT0. We minimized the number of animals used in each experiment (sample size based on power analysis) and the animals’ pain and distress. Mouse studies were conducted in accordance with regulations of the Institutional Animal Care and Use Committee at UofSC.

### Chronic light cycle disruption paradigm

**Figure 1:**
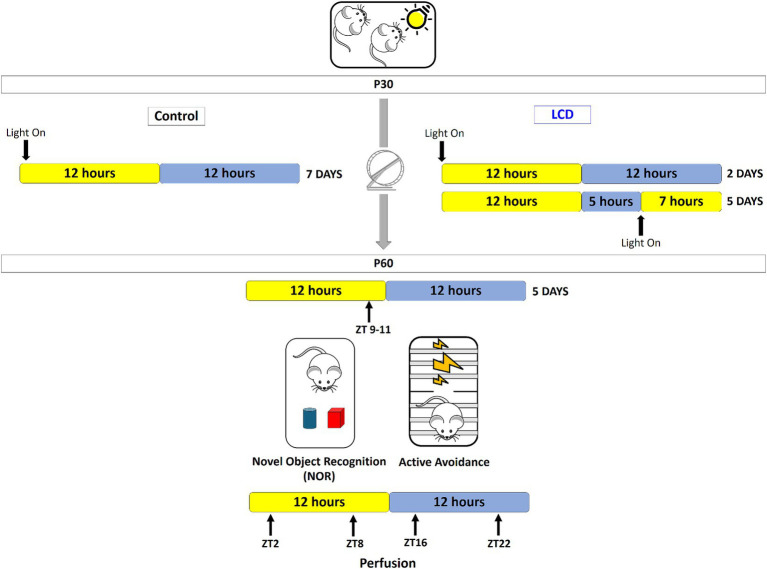
Novel light cycle disruption paradigm. Starting at P30, adolescent mice (post-natal day 30) were transferred into light-tight circadian cabinets and subjected to one of the following lighting conditions for a period of 4 weeks: Control conditions consisted in 12 h of light and 12 h of darkness (12 L:12D) per day for 7 days; for the LCD conditions LED bright light is turned on at the end of the active phase of the animals’ cycle and mice are exposed to an extended light phase of 19-h per day with LED light and 5 h darkness (19 L:5D) for 5 days followed by 12 L:12D for the next 2 days. Following the 4 weeks, at P60, mice were returned to standard 12 L:12D cycles for 5 days prior to testing novel object recognition and avoidance behavior. Behaviors were performed at the end of the light phase between ZT 9–11. 24 h after the behavioral test, brains were collected at 4 different ZT points (ZT2, ZT8, ZT16, and ZT22) for histological evaluation.

Adolescent mice (post-natal day 30) were transferred into light-tight circadian cabinets (Actimetrics, IL, United States) and subjected to one of the following lighting conditions for a period of 4 weeks: (1) standard 12:12 h light–dark cycles (12 L:12D) (light at ∼600 lux) (Control group); (2) 19 h of light and 5 h of darkness for 5 days and 12 L:12D for 2 days per week (LCD group) (Figure 1). LED light (~600 lux) is turned on during the night phase of the animals’ cycle, resulting in increased duration of light exposure for 5 days of a 7-day cycle. After 4 weeks of either LCD or control conditions, mice were placed in 12 L:12D for 5 days and tested for memory and avoidance behaviors and then sacrificed. Three cohorts of mice were used: one for assessing wheel-running activity, one for light pulse experiment and one for novel object recognition and active avoidance tests.

### Wheel running activity

Mice (post-natal day 20) were individually housed in running wheel–equipped cages, placed in light-proof circadian cabinets under controlled light intensity (∼600 lux) whose timing was controlled by an external timer. After a 10-day acclimatization, adolescent mice (post-natal day 30) were exposed to 4 weeks of either 12 L:12D (control) or LCD (19 L:5D;12 L:12D). Locomotor activity was monitored by continuously recording wheel revolutions and data was collected and analyzed using ClockLab software version 6 (Actimetrics, United States). The last 3 weeks of the experiment were analyzed for each condition. We calculated circadian activity variables including total wheel-running activity, daily rhythm amplitude, rhythm period, and wheel running activity onset. The total activity was calculated as the average number of wheel revolutions per 24 h over 3 weeks. Total and weekly period and total amplitude were calculated using a chi-square periodogram, activity was obtained with the daily countings and expressed as total activity and weekdays/weekend activity, and onset was also calculated for weekdays and weekends. Non-parametric circadian rhythm analysis were also performed including interdaily stability, which quantifies the invariability between the days, which gives an indication of the rhythm fragmentation, and relative amplitude, which quantifies the robustness of the rhythm (Van Someren et al., 1999). In addition, a detailed analysis of activity bouts was also done, with the term bout defined as a sustained period of activity. Specifically, we calculated the total number of activity bouts, the number of activity bouts per day, the average length of the activity bouts and the average peak rate of activity (defined as the maximum number of counts per minute).

### Novel object recognition

After 4 weeks of either LCD or control conditions, mice were placed in 12 L:12D for 5 days and tested on the novel object recognition task. The novel object recognition task is divided in 3 days: On day 1, mice were placed in an open arena (40 cm × 40 cm × 40 cm) and were allowed to explore for 5 min before being returned to their home cage. On day 2, mice were placed in the same open arena with two identical quadratic yellow blocks placed on the diagonal at an equal distance from the walls and were allowed to freely explore the objects for 10 min. Mice were then returned to their home cage. On day 3, mice were placed in the arena with the familiar object and a novel object which consisted of a quadratic red block and were allowed to explore both objects for 10 min. At the end of each test, the arena and objects were sanitized with 70% v/v ethanol. The habituation and the test days were conducted at ZT9-11. Mice were recorded using Ethovision software, and the time exploring the familiar and the novel object was calculated as the percentage of time spent with the novel versus the familiar object. We also analyzed the discrimination index (DI), calculated as the time spent exploring the novel object minus the time spent exploring the familiar object divided by total exploration time. A value below zero describes subjects exploring the familiar object more than the novel object. A value above zero describes animals exploring the novel object more than the familiar object.

### Active avoidance

After 4 weeks of either LCD or control conditions, mice were placed in 12 L:12D for 5 days and transferred to a shuttle box (San Diego Instruments, San Diego, CA, United States) at ZT9-11. Mice received 30 electric foot shocks through the grid floor. During each test shock (0.10 mA, maximum duration 30 s) the gate between the two compartments remained open, and mice had a chance to escape the shock by crossing to the adjacent compartment. The schedule in trials #1–5 was fixed ratio (FR)-1 (crossing the gate once in order to escape the shock). In the remaining trials #6–30, the schedule was changed to FR-2 (crossing the gate twice in order to escape the shock). The shock was omitted when the animals crossed the gate the appropriate number of times. The number of escape failures and the escape latency were used as a measure of the avoidance responses, as previously described (López-Moraga et al., 2022). Only the C57BL/6 J mice were used for this test since the foot shock intensity protocol is based on C57BL/6 J sensitivity (Landgraf et al., 2015).

### Immunohistochemistry

After 4 weeks of either LCD or control conditions, mice were placed in 12 L:12D for 5 days and then sacrificed at four different ZTs: (in reference to the daily light cycle): ZT2, ZT8, ZT16, and ZT22. Mice were deeply anesthetized with isoflurane and transcardially perfused with 1% phosphate-buffered saline (PBS) solution immediately followed by 4% paraformaldehyde solution in PBS (pH = 7.4) for tissue fixation. Then, the brains were collected, post-fixed in 4% PFA for 24 h, cryoprotected in 30% sucrose solution, and stored at 4°C until use. Frozen brains were sectioned (30 μm) with a standard Leica microtome (SM2010R) and stored in cryoprotectant solution at −20°C until use. Six to seven coronal sections from each brain encompassing the SCN, MeA, and hippocampus were processed for immunohistochemistry (IHC) analysis. Immunofluorescent labeling was performed in free-floating slices that were first blocked for 1 h in 0.1 M phosphate buffer (PB) containing 3% normal horse serum (NHS) and then permeabilized in 0.2% Triton X-100. Slices were then incubated with antibodies: rabbit anti-SST (1:1000; PA5-85759, Invitrogen, United States), goat anti–c-FOS (1:500; SC-52-G Santa Cruz, USA) in 1% PBS, 3% NHS, and 0.2% Triton X-100 for 24 or 48 h at 4°C with constant shaking. After three washes in PBS, slices were incubated for 1 h at room temperature with fluorescent-tagged secondary antibodies, which were all used at 1:1,000 dilution. (1:100; AlexaFluor anti-rabbit 647 nm, anti-goat pig 555 nm). Brain sections were washed, mounted in gelatin on glass slides, counter-stained, and cover-slipped with Dapi Fluoromount-G™ (Electron Microscopy Sciences, United States). Images were taken with a Leica TCS SP8 multiphoton confocal microscopy system (Leica Microsystems, Germany) and LASX software (Leica Microsystems, Germany). Neuronal quantifications were performed blind with ImageJ software. Quantification data were plotted either as the average or as the total number of positive neurons per nucleus per animal.

### Rnascope^®^ *in situ* hybridization

For each mouse, two to three slices encompassing the SCN, MeA and hippocampus were processed for RNAscope *in situ* hybridization [Multiplex Fluorescent Reagent Kit version 2, Advanced Cell Diagnostics (ACD)] following the manufacturer’s instructions for detection of VGAT (ACD, catalog #319191), VGLUT2 (ACD, catalog #319171), SST (ACD, catalog #404631), Per1 (ACD, catalog #438751), Clock (ACD, catalog #492401) and c-FOS (ACD, catalog #316921) mRNA at 4 different ZTs: ZT2, ZT8, ZT16, and ZT22. Sections were washed in PBS, treated with hydrogen peroxide and mounted in glass slides with 1% PBS. Later, brain sections were incubated in the retrieval buffer and dehydrated in ethanol, previously to a protease treatment. Then, sections were incubated with the specific probes for 2 h at 40°C, and the amplification steps were followed as indicated by the manufacturers. Finally, sections were counter-stained, and cover-slipped with Dapi Fluoromount-G™ (Electron Microscopy Sciences, United States). Images were taken with a Leica TCS SP8 multiphoton confocal microscopy system (Leica Microsystems, Germany) and LASX software (Leica Microsystems, Germany). Quantifications were performed blind with Fiji ImageJ software. Semiquantitative histological scoring methodology was used to quantify clock genes Period1 (Per1) and Clock. A score of 0 means no staining or less than 1 dot for every neuron, whereas a score of 4 means greater than 15 dots per neuron/cell or > 10% dots in clusters.

### Light pulse experiment

Light pulse experiments were performed as previously described (Fernandez et al., 2018). Adolescent mice housed under 12 L:12D conditions were kept in constant darkness (DD) for 24 h to avoid any light exposure effect. Mice were exposed to a 30 min, 600 lux light pulse light at the beginning of the subjective night (CT14) and then transferred back in DD for an additional 45 min. Mice were then perfused with 4% paraformaldehyde, and brains were collected as described in the Immunohistochemistry paragraph. Control mice were kept in DD and sacrificed at the same CT.

### Statistical analysis

Investigators who participated in end point analyses were blinded to the light protocol. Statistical analysis was carried out using GraphPad Prism (GraphPad Software, La Jolla, CA, United States). For rhythmicity analysis, data were fitted to a sinusoidal curve using the nonlinear least-squares regression comprising both sine and cosine waveforms (constrained period of 24 h). The coefficient of determination (R^2^) was used as a proxy of goodness-of-fit. Additionally, the empirical *p* value was calculated using CircWave v.1.4. Outliers were identified and removed by the ROUT method provided by Graph Pad Prism Software, and normality was assessed (Shapiro–Wilk, Kolmogorov–Smirnov, D’Agostino and Pearson, and Anderson–Darling tests) before performing the corresponding statistical analyses. For normally distributed data, a parametric test was used one-way or two-way analysis of variance (ANOVA) or Student’s *t*-test and *p*-value less than 0.05 was considered significant, and was indicated as followed: *p < 0.05; **p < 0.01; ***p < 0.001; ****p < 0.0001. The statistical tests performed for each experiment are indicated in the figure legends.

## Results

### LCD does not alter circadian rhythms of locomotor activity in adolescent mice

**Figure 2:**
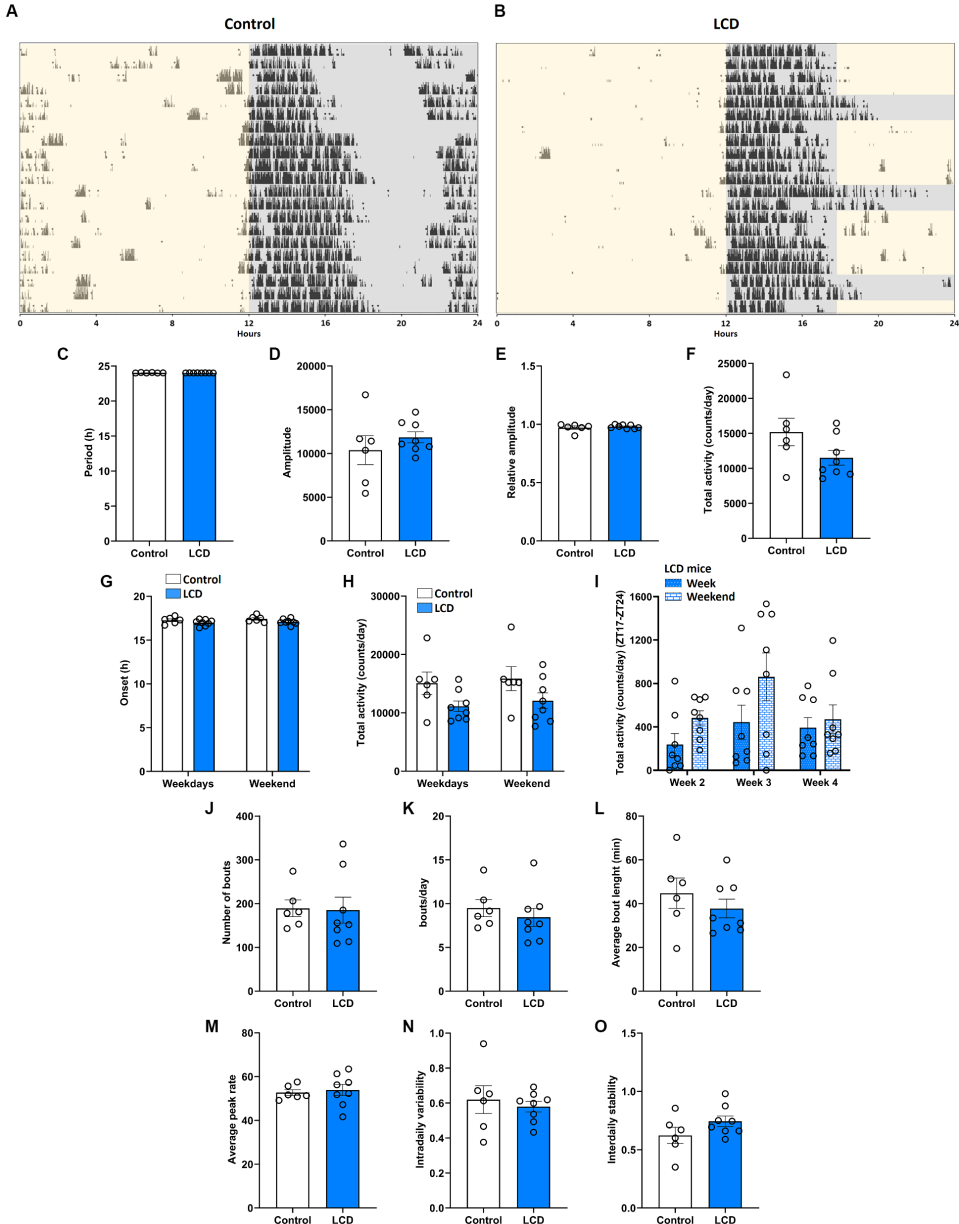
Effect of LCD on locomotor activity rhythms. Wheel running activity analysis under control and LCD conditions for 4 weeks on adolescent mice. Representative actograms for each lighting condition are shown: **(A)** Control and **(B)** LCD. The light phase is represented with a yellow background and the dark phase with a gray background. Variables were analyzed in the last 3 weeks of each condition, including **(C)** period, **(D)** amplitude, **(E)** relative amplitude, **(F)** total activity counts, **(G)** onset during the weekdays and weekends [*F*_(1, 24)_ = 5.156, *p* < 0.0324 by two-way ANOVA with Šídák’s multiple comparison posttest], **(H)** total activity counts during the weekdays and weekends [*F*_(1, 24)_ = 6.469, *p* < 0.0178 by two-way ANOVA with Šídák’s multiple comparison posttest], **(I)** total activity counts in LCD mice during the weekdays and weekends [*F*(1, 42) = 4.821, *p* < 0.0337 by two-way ANOVA with Šídák’s multiple comparison posttest], **(J)** number of bouts, **(K)** bouts per day, **(L)** average bout length, **(M)** average peak rate, **(N)** intradaily variability and **(O)** interdaily stability,. Individual data points represent independent mice, data are shown as mean ± SEM. (Control*n* = 3 females and *n* = 3 males; LCD *n* = 4 females, *n* = 4 males). Student’s t-test: (with Welch’s correction when appropriate); two-way ANOVA (post-hoc test conducted with Šídák’s multiple comparison test).

We first evaluated the effect of LCD exposure on the circadian locomotor activity rhythms using voluntary wheel-running activity (Figure 2). Running wheel activity of singly housed mice was recorded in control and LCD condition for 4 weeks, as shown in Figures 2A,B. Wheel-running analysis revealed that control and LCD mice exhibited similar period (h) (Figure 2C), amplitude (Figure 2D), relative amplitude (Figure 2E) and total activity (Figure 2F). Analysis also revealed similar activity onsets and total activity within the weekdays and weekends (Figures 2G,H respectively). We further analyzed the total activity from ZT17 to ZT24 in LCD mice (Figure 2I) and found no significant differences when comparing the effect of 7 h of light exposure on weekdays versus the dark phase during the weekends. The activity bouts were analyzed to evaluate the fragmentation of the activity during the day. No significant differences were found in the total number of bouts (Figure 2J), bouts per day (Figure 2K), average bout length (Figure 2L) and average peak rate (Figure 2M) between light conditions. Finally, non-parametric analysis showed no significant differences in the intradaily variability (Figure 2N) and interdaily stability (Figure 2O) between control and LCD mice. Altogether, these results indicate that exposure to LCD has no effect on the circadian wheel-running activity in adolescent mice.

### LCD does not alter circadian gene expression and neuronal activity rhythms in the SCN

**Figure 3:**
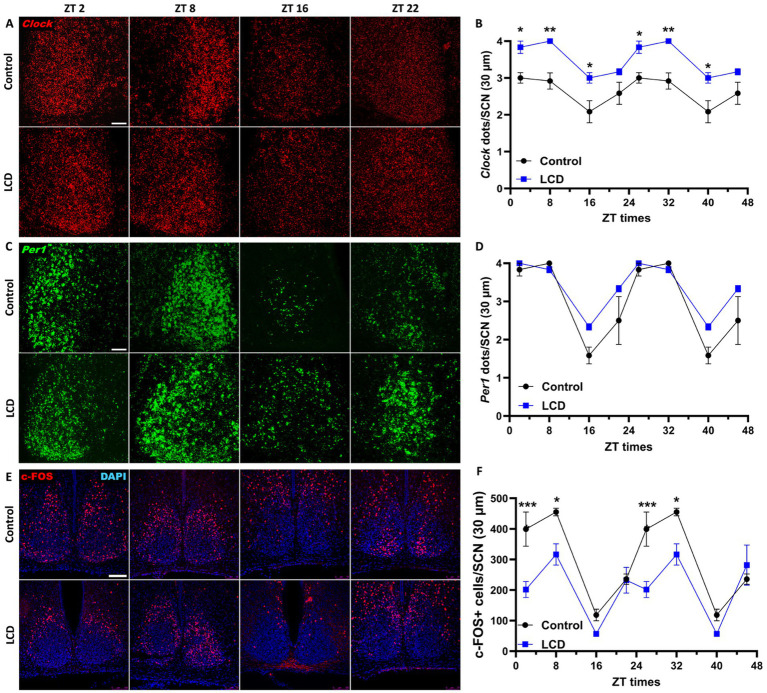
Daily expression of Per1, Clock and cFOS in the SCN. Representative confocal micrographs showing **(A)** *Clock* (red) and **(C)** *Per1* (green) mRNA expression detected by RNAscope and **(E)** c-FOS (red) detected by immunofluorescence at ZT2, ZT8, ZT16 and ZT22 in control and LCD mice (Scale bar 50 μm). Line graphs show **(B)** *Clock* mRNA expression (F_1,32_ = 76.41, *p* < 0.0001 by two-way ANOVA with Šídák’s multiple comparison posttest) and **(D)** *Per1* mRNA expression [*F*_(1, 32)_ = 10.17, *p* < 0.0032 by two-way ANOVA with Šídák’s multiple comparison posttest] determined by semiquantitative scoring of Clock and Per1 dots and clusters per neuron, and **(F)** number of c-FOS positive neurons in the SCN [*F*_(1, 32)_ = 38.01, *p* < 0.0001 by two-way ANOVA with Šídák’s multiple comparison posttest]. Data are shown as mean ± SEM. (Control *n* = 2 females and *n* = 2 males; LCD *n* = 2 females, *n* = 2 males) for the SCN in a 30-μm section; *p < 0.05, **p < 0.01, ***p < 0.001, ****p < 0.0001, two-way ANOVA (*post-hoc* test conducted with Šídák’s multiple comparison test).

Exhaustive research previously showed that altered light environments disrupt the molecular circadian clock in the SCN along with the circadian locomotor activity (Shuboni and Yan, 2010; Fonken et al., 2013; Ikeno and Yan, 2016). Although our wheel-running activity analysis did not reveal any effect of the LCD on the circadian locomotor rhythms, we evaluated whether LCD exposure altered the expression patterns of *Clock* and *Per1* genes in the SCN. Thus, adolescent mice exposed to either control or LCD for 4 weeks were sacrificed at 4 different ZTs: ZT2, ZT8, ZT16, and ZT22. Brain sections encompassing the SCN were then assessed for *Clock* and *Per1* expression using *in situ* hybridization (ISH) RNAscope. Both *Clock* and *Per1* showed significant rhythmicity in control and LCD mice (Supplementary Figures S1A,B). We found significantly increased expression of *Clock* gene at ZT2 and ZT16 in LCD mice compared to control (Figures 3A,B), with no change in the daily expression patterns. No differences were observed in *Per1* expression across the 24 h cycle (Figures 3C,D). To determine the effect of LCD on neuronal activity rhythm in the SCN, c-FOS, a proxy for neuronal activity, was quantified at ZT2, ZT8, ZT16, and ZT22. We found that c-FOS expression shows significant rhythmicity in control and LCD mice in the SCN (Supplementary Figure S1C). Interestingly, LCD mice show a significant reduction in the number of c-FOS positive cells during the light phase at ZT2, and ZT8 (Figures 3E,F). Together, these results revealed that LCD exposure during adolescence does not alter circadian clock expression and neuronal activity rhythms in the SCN.

### LCD impairs memory in the NOR test

**Figure 4:**
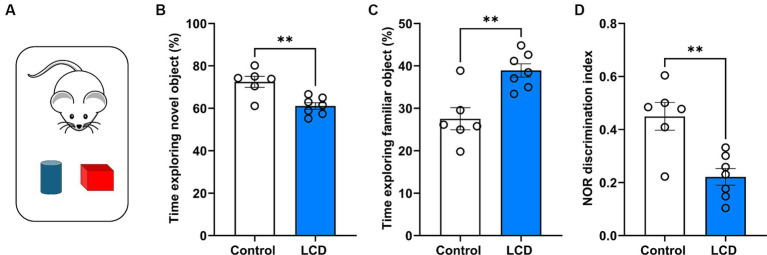
Effects of LCD on novel object recognition. **(A)** Schematic representation of the NOR test day 3 showing the mice with the familiar (blue) and the novel object (red). Bar graphs show the percentage of time spent exploring **(B)** the novel object, **(C)** the familiar object and **(D)** the discrimination index. Individual data points represent independent mice. Data are shown as mean ± SEM. (Control *n* = 3 females and *n* = 3 males; LCD *n* = 4 females, *n* = 4 males). **p < 0.01, Student’s *t*-test (with Welch’s correction when appropriate).

Given recent evidence showing impaired memory and cognition in rodents after exposure to altered light environments (Liu et al., 2022; Sangma and Trivedi, 2023), we evaluated whether exposure to LCD alters long-term memory using the NOR task (Lueptow, 2017) (Figure 4A). We found that mice exposed to the LCD showed decreased time exploring the novel object (Figure 4B) and increased time exploring the familiar object (Figure 4C) compared to the control group. Less exploration of the novel object and more exploration of the familiar object led to a significantly lower discrimination index for the LCD mice in comparison to the control group (Figure 4D). Altogether, these results show that exposure to LCD during adolescence impairs object recognition long-term memory.

### LCD does not affect daily Clock and Per1 expression patterns but induces a phase advance in the neuronal activity rhythm in the dentate gyrus

**Figure 5:**
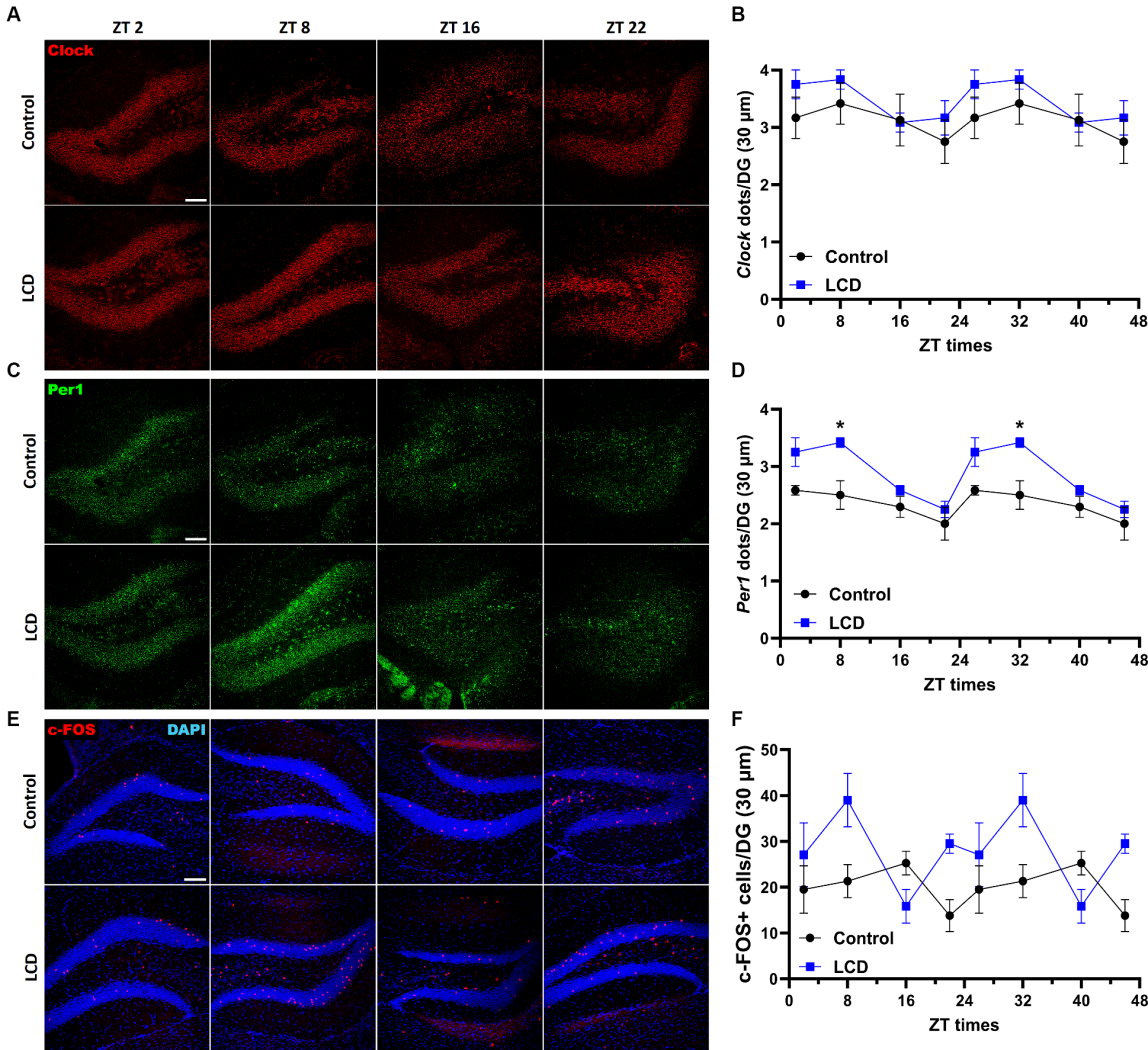
Daily expression of Per1, Clock and cFOS in the Dentate Gyrus. Representative confocal micrographs showing **(A)** *Clock* (red) and **(C)** *Per1* (green) mRNA expression detected by RNAscope and **(E)** c-FOS (red) detected by immunofluorescence at ZT2, ZT8, ZT16 and ZT22 in control and LCD mice (Scale bar 100 μm). Line graphs show **(B)** *Clock* mRNA expression [*F*_(1, 32)_ = 4.591, *p* < 0.0398 by two-way ANOVA with Šídák’s multiple comparison posttest] and **(D)** *Per1* mRNA expression [*F*_(1, 32)_ = 31.80, *p* < 0.0001 by two-way ANOVA with Šídák’s multiple comparison posttest] determined by semiquantitative scoring of *Clock* and *Per1* dots and clusters per neuron, and **(F)** number of c-FOS positive neurons in the DG [*F*_(1, 38)_ = 11.51, *p* < 0.0016 by two-way ANOVA with Šídák’s multiple comparison posttest]. Data are shown as mean ± SEM. (Control *n* = 2 females and *n* = 2 males; LCD *n* = 2 females, *n* = 2 males) for the DG in a 30-μm section; *p < 0.05, two-way ANOVA (*post-hoc test* conducted with Šídák’s multiple comparison test).

The dentate gyrus (DG) of the hippocampus regulates object recognition in rodents (Jessberger et al., 2009; Dees and Kesner, 2013; Jiang et al., 2023) and exposure to altered light environments induces memory impairment in rats (Sangma and Trivedi, 2023). Furthermore, the DG displays clock genes rhythmicity regulating memory (Jilg et al., 2010; Gonzalez et al., 2023). Hence, we analyzed *Clock* and *Per1* expression patterns and neuronal activity rhythms using c-FOS in the DG at ZT2, ZT78, ZT16, and ZT22. No differences were found in both *Clock* and *Per1* daily expression patterns and rhythmicity (Figures 5A–D; Supplementary Figures S1D,E). However, we found that LCD mice showed a significant increase in *Per1* expression at ZT8 compared to control (Figures 5C,D). Immunofluorescence analysis of c-FOS expression in the DG revealed no significant rhythmicity in the neuronal activity in LCD or in control mice (Supplementary Figure S1F). Nevertheless, we observed a phase advance in neuronal activity rhythm as shown by increased c-FOS expression at ZT8 in LCD mice compared to control mice (Figures 5E,F). These data show that despite small alterations in clock genes’ expression patterns, LCD altered the neuronal activity rhythm in the granule layer of the DG.

### LCD mice show impaired avoidance behavior

**Figure 6:**
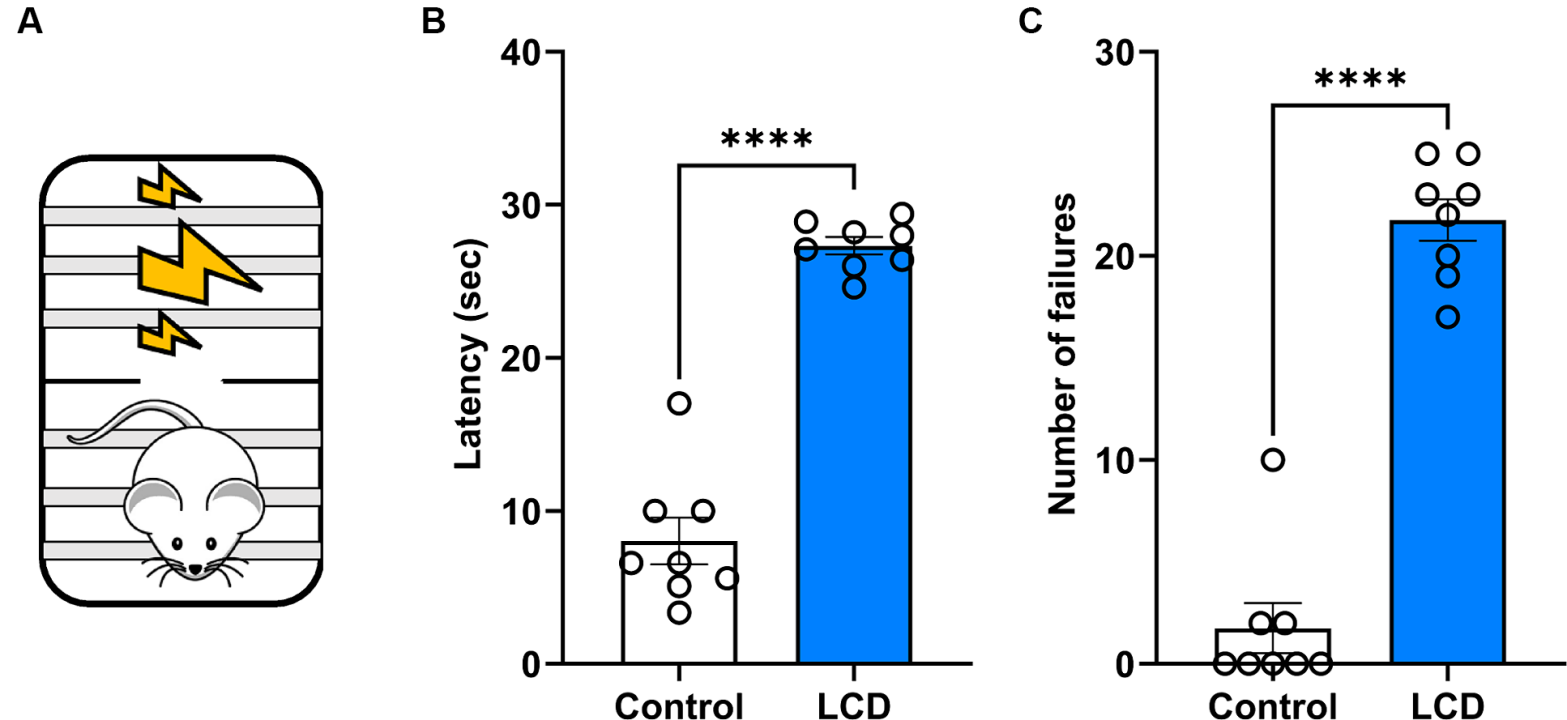
Effects of LCD on active avoidance. **(A)** Schematic representation of the active avoidance test. Bar graphs showing **(B)** the Latency and **(C)** the number of failures in the active avoidance test. Individual data points represent independent mice, data are shown as mean ± SEM. (Control *n* = 4 females and *n* = 4 males; LCD *n* = 4 females, *n* = 4 males). ****p < 0.0001, Student’s *t*-test (with Welch’s correction when appropriate).

Previous investigations showed altered affective behaviors following aberrant light exposure (LeGates et al., 2012; Fernandez et al., 2018). Here, we studied the effect of exposure to LCD during adolescence on avoidance behavior using an active avoidance test (Figure 6A). We found that LCD mice showed a significant increase in the latency (Figure 6B) and in the number of failures (Figure 6C) to escape the foot shock compared to control mice. These data suggest that LCD exposure during adolescence increases avoidance responses, such as freezing behavior in response to an aversive stimulus.

### LCD disrupts Clock and Per1 expression patterns and induces a phase advance in neuronal activity rhythm in the MeA

**Figure 7:**
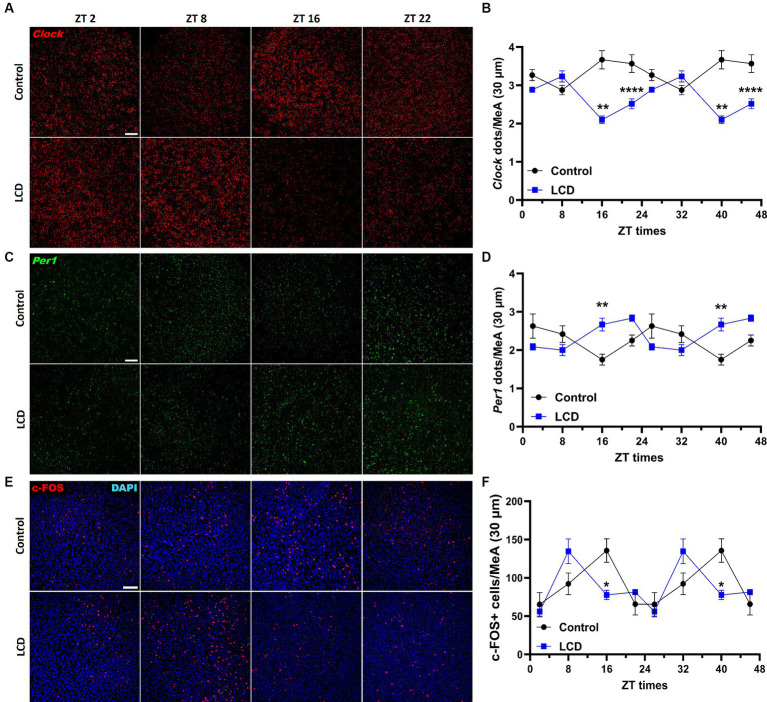
Daily expression of Per1, Clock and cFOS in the MeA. Representative confocal micrographs showing **(A)** *Clock* (red) and **(C)** *Per1* (green) mRNA expression detected by RNAscope and (Scale bar 50 μm) **(E)** c-FOS (red) (Scale bar 100 μm) detected by immunofluorescence at ZT2, ZT8, ZT16 and ZT22 in control and LCD mice. Line graphs show **(B)** *Clock* mrNA expression [*F*_(1, 32)_ = 72.74, *p* < 0.0001 by two-way ANOVA with Šídák’s multiple comparison posttest] and **(D)** *Per1* mRNA expression [*F*_(1, 32)_ = 2.313, *p* < 0.1381 by two-way ANOVA with Šídák’s multiple comparison posttest] determined by semiquantitative scoring of *Clock* and *Per1* dots and clusters per neuron, and **(F)** number of c-FOS positive neurons in the MeA [*F*_(1, 34)_ = 0.1398, *p* < 0.7108 by two-way ANOVA with Šídák’s multiple comparison posttest]. Data are shown as mean ± SEM. (Control *n* = 2 females and *n* = 2 males; LCD *n* = 2 females, *n* = 2 males) for the MeA in a 30-μm section; * *p*<0.05. **p < 0.01, ****p < 0.0001, two-way ANOVA (*post-hoc* test conducted with Šídák’s multiple comparison test).

Although the MeA receives direct light inputs (Hattar et al., 2006) and is involved in the regulation of avoidance behaviors (Miller et al., 2019), no study has been conducted to unveil alterations in the MeA induced by altered light environments. Therefore, we first assessed the effect of LCD exposure on the MeA molecular clock by quantifying *Clock* and *Per1* mRNA expression at ZT2, ZT8, ZT16, and ZT22 using RNAscope. Both *Clock* and *Per1* exhibit significant rhythmicity in the MeA of control and LCD mice (Supplementary Figures S1G,H). However, we found that LCD significantly altered *Clock* and *Per1* expression in the MeA, as shown by their antiphase expression patterns compared to control mice (Figures 7B,D). Specifically, LCD induced a significant reduction in *Clock* expression during the dark phase at ZT16 and ZT22 (Figures 7A,B) and a significant increase of *Per1* expression at ZT16 compared to control (Figures 7C,D). To test the effect of LCD on neuronal activity rhythms in the MeA, we analyzed c-FOS expression at 4 ZT times. We found that control and LCD mice show significant neuronal activity rhythmicity (Supplementary Figure S1I), and that LCD induced a phase advance in neuronal activity rhythm compared to control (Figures 7E,F). Thus, while control mice display a peak of c-FOS expression at ZT16, mice exposed to LCD show a peak of c-FOS at ZT8 followed by a reduction at ZT16 (Figures 7E,F). Altogether, these data show that LCD exposure during adolescence disrupts the MeA molecular clock and the neuronal activity rhythm.

### A 30-min LED light pulse at night alters Per1 expression and neuronal activity in somatostatin neurons in the MeA

**Figure 8:**
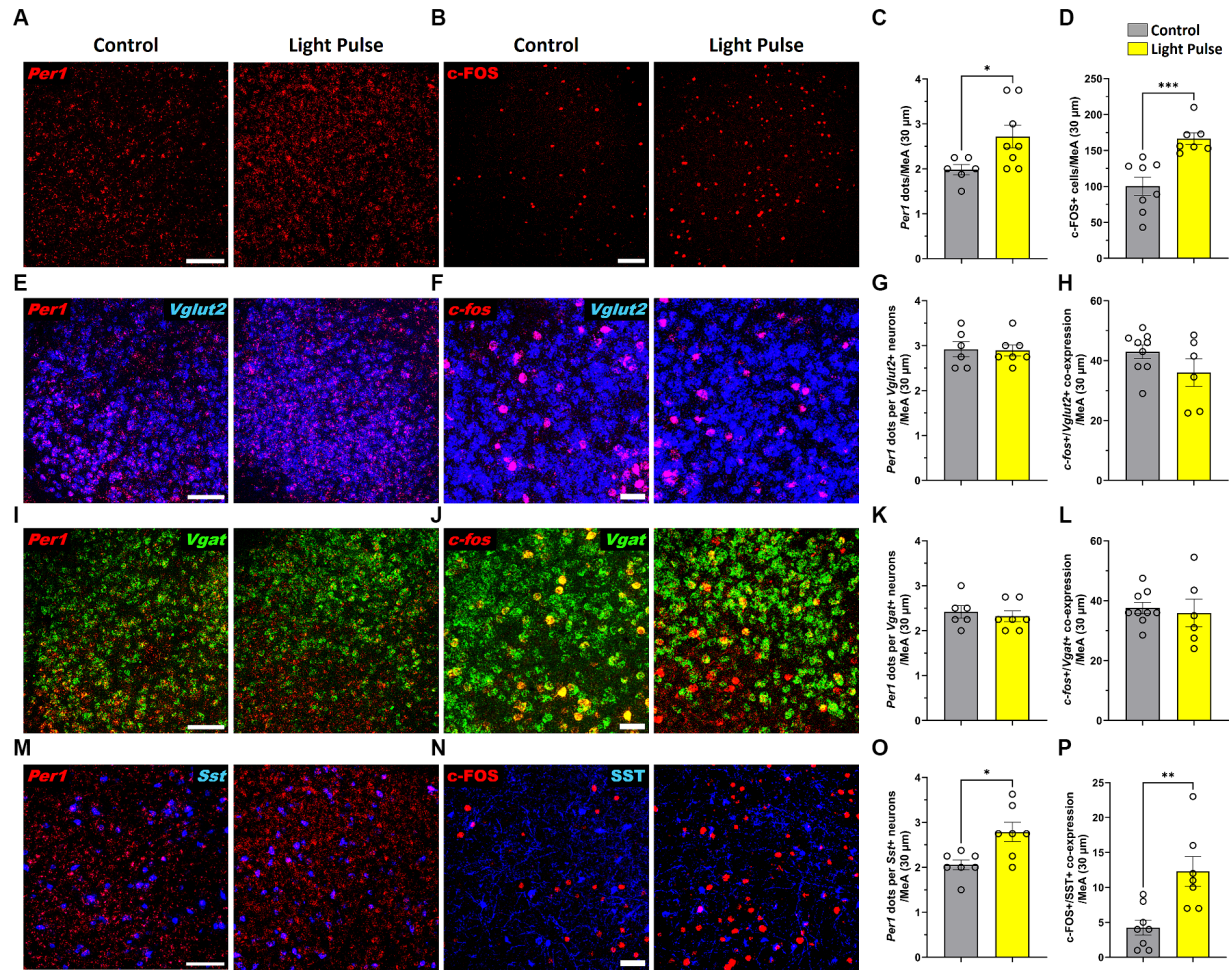
Representative confocal micrographs showing **(A)** *Per1* (red) mRNA expression detected by RNAscope and **(B)** c-FOS (red) detected by immunofluorescence (Scale bar 100 μm) in the MeA; Representative confocal micrographs showing **(E)** *Per1* (red) **(F)** and *c-fos* (red) mRNA expression detected by RNAscope in glutamatergic neurons (*VGLUT2*, blue) (Scale bar 100 μm); Representative confocal micrographs showing **(I)** *Per1* (red) **(J)** and *c-fos* (red) mRNA expression detected by RNAscope in GABAergic neurons (*VGAT*, green); Representative confocal micrographs showing **(M)** *Per1* (red) mRNA expression detected by RNAscope (Scale bar 100 μm) and **(N)**) c-FOS (red) (Scale bar 50 μm) detected by immunofluorescence in SST neurons (SST, blue). Bar graphs show *Per1* mRNA expression determined by semiquantitative scoring of *Per1* dots and clusters per neuron in the **(C)** MeA, **(G)** glutamatergic neurons, **(K)** GABAergic neurons and **(O)** SST neurons in the MeA; and c-FOS/*c-fos* expression in the **(D)** MeA, **(H)** glutamatergic neurons, **(L)** GABAergic neurons and **(P)** SST neurons in the MeA. Individual data points represent independent mice, data are shown as mean ± SEM. (Control *n* = 3 females and *n* = 3 males; LCD *n* = 4 females, *n* = 4 males). *p < 0.05, **p < 0.01, ***p < 0.001, Student’s *t*-test (with Welch’s correction when appropriate).

To confirm photoresponsiveness of MeA neurons during adolescence, adolescent mice housed in 12 L:12D condition were placed in DD for 24 h and then exposed to a single 30-min light pulse at the beginning of their subjective dark phase (Circadian time CT14) and then sacrificed. MeA photoresponsiveness was evaluated by analyzing *Per1* and c-FOS expression using RNAscope and immunofluorescence. We found that a 30-min light pulse significantly increased *Per1* and c-FOS expression in the MeA (Figures 8A–D). Since the MeA is composed of heterogeneous neuronal subpopulations (Wu et al., 2017), we specifically analyzed the acute effect of light at night in glutamatergic and GABAergic neurons. We found that a 30-min light pulse did not affect *Per1* and *c-fos* expression in glutamatergic (Figures 8E–H) nor in GABAergic populations in the MeA (Figures 8I–L). Considering the role of somatostatin (SST) in the amygdala in the circadian modulation of anxiety (Albrecht et al., 2013), we assessed *Per1* and c-FOS expression in SST neurons in the MeA. We found that a 30-min light pulse at night induced a significant increase of *Per1* and c-FOS expression in the SST neuronal subpopulation in the MeA (Figures 8M–P) compared to control mice. Our analysis revealed for the first time that selective subtypes of MeA neurons are affected by acute exposure to light at night.

### LCD disrupts the molecular clock in SST neurons and SST neuronal activity rhythm but exerts no effect on the SST expression patterns

**Figure 9:**
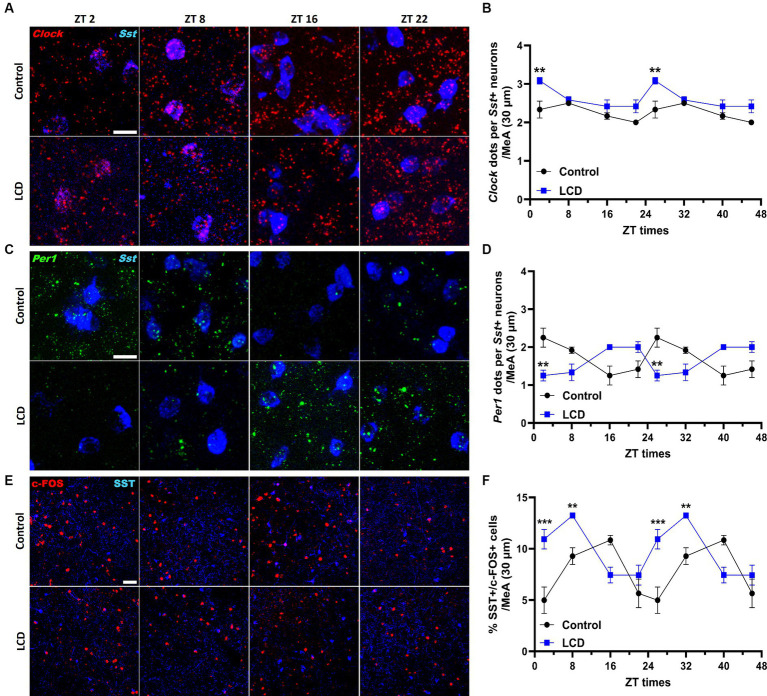
Daily expression of Per1, Clock and cFOS in the SST neurons of the MeA. Representative confocal micrographs showing **(A)** *Clock* (red) and **(C)** *Per1* (green) mRNA expression detected by RNAscope and (Scale bar 50 μm) **(E)** c-FOS (red) (Scale bar 100 μm) detected by immunofluorescence at ZT2, ZT8, ZT16, and ZT22 in control and LCD mice. Line graphs show **(B)** *Clock* mRNA expression [*F*_(1, 32)_ = 36, *p* < 0.0001 by two-way ANOVA with Šídák’s multiple comparison posttest] and **(D)** *Per1* mRNA expression [*F*_(1, 32)_ = 0.4615, *p* < 0.5018 by two-way ANOVA with Šídák’s multiple comparison posttest] determined by semiquantitative scoring of Clock and Per1 dots and clusters per neuron, and **(F)** number of c-FOS positive neurons in the MeA [*F*_(1, 32)_ = 0.1398, *p* < 0.7108 by two-way ANOVA with Šídák’s multiple comparison posttest]. Data are shown as mean ± SEM. (Control *n* = 2 females and *n* = 2 males; LCD *n* = 2 females, *n* = 2 males) for the MeA in a 30-μm section; **p < 0.01, ***p>0.001, ****p < 0.0001, two-way ANOVA (*post-hoc* test conducted with Šídák’s multiple comparison test).

**Figure 10:**
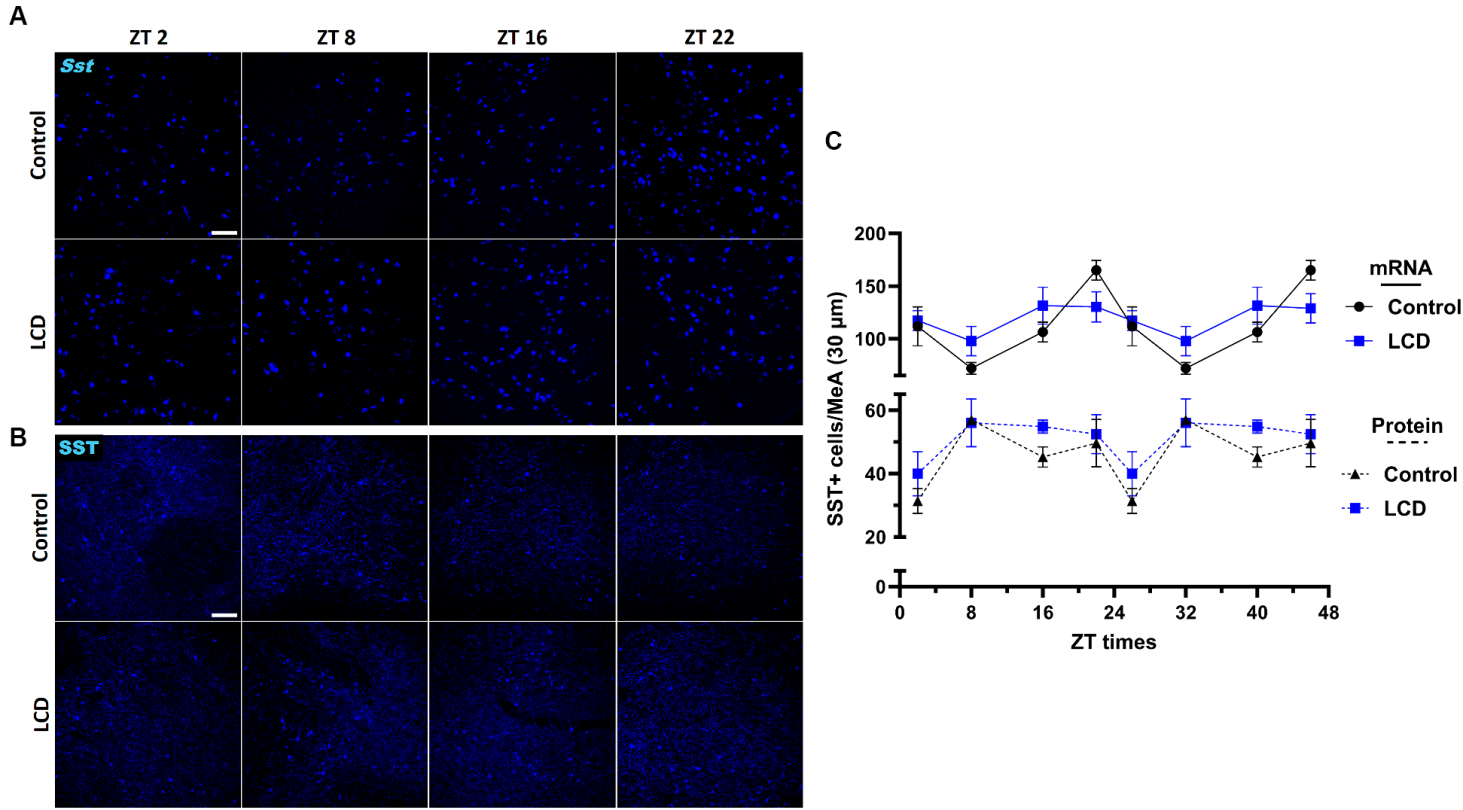
Daily expression of SST mRNA and protein in the MeA. Representative confocal micrographs showing **(A)** Sst (blue) mRNA expression detected by RNAscope and **(B)** SST (blue) protein expression detected by immunofluorescence at ZT2, ZT8, ZT16 and ZT22 in control and LCD mice (Scale bar 100 μm). **(C)** Line graph shows number of Sst+ mRNA expressing neurons (solid line), and **(D)** number of SST+ protein expressing neurons (dotted line) in the MeA. Data are shown as mean ± SEM (Control *n* = 2 females and *n* = 2 males; LCD *n* = 2 females, *n* = 2 males) for the MeA in a 30-μm section; Two-way ANOVA (Post-hoc test conducted with Šídák’s multiple comparison test).

Given that SST in the amygdala plays a crucial role in mediating avoidance behavior (Yu et al., 2016) we hypothesized that LCD influences clock gene expression and neuronal activity rhythms in SST neurons, consequently impacting associated behaviors. Thus, we investigated the effect of LCD exposure on *Clock* and *Per1* expressions at ZT2, ZT8, ZT16, and ZT22 in SST neurons. We found that LCD exposure disrupted *Clock* rhythmicity (Supplementary Figure S1J) and increased the overall daily *Clock* expression with a significant increase at ZT2 compared to control mice (Figures 9A,B). Mice exposed to LCD displayed anti-phasic *Per1* expression patterns compared to control mice (Figures 9C,D), showing significant differences at ZT2 with no changes in rhythmicity (Supplementary Figure S1K). We further evaluated SST neuronal activity rhythm by measuring c-FOS expression by immunofluorescence. We found that control and LCD mice show significant SST neuronal activity rhythmicity (Supplementary Figure S1L) and that LCD induced a phase advance in SST neuronal activity rhythm compared to control, showing a peak of c-FOS at ZT8 and a significant reduction at ZT16, while control mice showed a peak at ZT16 (Figures 9E,F). Considering the role of SST expression in the amygdala on avoidance behavior and in modulation of anxiety (Albrecht et al., 2013; Ahrens et al., 2018; Sun et al., 2020), we evaluated the effect of LCD exposure on SST expression in the MeA by RNAscope and immunofluorescence at ZT2, ZT8, ZT16, and ZT22. We found no difference in the SST expression patterns neither at the mRNA nor at the protein levels between control and LCD mice (Figures 10A–C). Overall, these data suggest that LCD disrupts molecular clock and neuronal activity patterns in the SST neurons in the MeA while not affecting daily SST expression.

## Discussion

Previous investigations have demonstrated a direct neural pathway through which light regulates affective behaviors independent of the circadian pacemaker (LeGates et al., 2012; Fernandez et al., 2018). A recent study in mice has implicated the perihabenular nucleus in mediating helpless behaviors induced by a fast ultradian photoperiod (Fernandez et al., 2018). However, such a fast light/dark cycle typically causes free-running of the circadian rhythm, so that in this T7 protocol, light exposure might occur at variable circadian times. While another recent study has demonstrated the impact of aberrant light exposure on amygdala circuits and avoidance behavior in adult mice (Wang et al., 2023), it is essential to note that the exposure duration in this study was relatively brief (30 min). Consequently, the relevance of these findings to humans, who commonly experience chronic exposure to altered light environments, may be limited.

Given the alterations in timing and duration of daily light exposure in human adolescents, developing preclinical models to study the impact of altered light environments during this crucial developmental stage is needed. In the current study, we developed a novel light protocol for chronic light cycles disruption that mimics changes in light/dark cycles experienced by adolescents. In this paradigm, adolescent mice are exposed to 19 L:5D light for 5 days and 12 L:12D for 2 days, for a total of 4 weeks. We found that mice exposed to LCD during adolescence exhibited impaired memory and avoidance behavior while showing no changes in circadian locomotor activity compared to control mice. Histological analysis revealed altered clock gene expression patterns and neuronal activity rhythms in brain regions regulating memory and emotional responses, such as the hippocampus and the MeA. No effects were observed in the SCN. Finally, our investigation unveiled for the first time photic responsiveness of MeA neuronal subpopulation and elucidated how exposure to LCD influences SST neurons. Altogether, our data provide a potential new mechanism by which alterations in the light environment impact behaviors during adolescence.

Wheel-running activity and histological analysis in the SCN revealed no difference in circadian locomotor activity and *Per1* and *Clock* expression in LCD mice compared to control mice suggesting preserved circadian properties of the SCN. Given that, the impairment of memory and avoidance behavior observed in LCD mice might be due to a SCN-independent pathway. Prior studies have shown that the consequences of light at night, such as enhanced T-cycle entrainment and behavioral rhythm bifurcation, cannot be solely attributed to simple masking (Gorman and Elliott, 2003; Walbeek and Gorman, 2017; Noguchi et al., 2020). Moreover, the behavioral effects of light at night often exhibit categorical patterns rather than modest adjustments in entrainment parameters according to bright light entrainment theory (Foster et al., 2020). Taken together, our results and previous findings demonstrate that under appropriate conditions, the circadian system exhibits greater flexibility than conventional circadian theory predicts (Walbeek et al., 2021).

The hippocampus is a subordinate circadian oscillator where more than 10% of genes and proteins show circadian fluctuations and are associated with changes in synaptic and neuronal excitability (Barnes et al., 1977; Debski et al., 2017; Gonzalez et al., 2023). Moreover, a number of studies show that altered light environment exposure induces memory impairment by increased oxidative stress and inflammation in the hippocampus in mice (Liu et al., 2022). Previously, Delorme demonstrated that long-term exposure to altered light environments increased the number of spines in hippocampal neurons (Delorme et al., 2022), suggesting an effect of light on neuroplasticity. Conversely, a previous study performed in adolescent mice revealed that dim light at night decreased spatial memory and hippocampal neurogenesis while increasing hippocampal oxidative stress (Namgyal et al., 2020). In addition, some studies also suggest that acute (Loh et al., 2010) and chronic (Gibson et al., 2010) jet lag leads to cognitive function deficits. Clock genes show circadian rhythmicity in the hippocampus, including the DG (Jilg et al., 2010), which is the area regulating novel object recognition memory. While *Per1* and *Clock* expression patterns were preserved in DG after LCD, we observed a phase advance in the neuronal activity rhythm in the granule layer of the DG. Previous studies showed that disrupted diurnal regulation of hippocampal inhibitory transmission altered cognition (Fusilier et al., 2021). Hence, LCD might impair novel object recognition memory by alteration of neuronal physiology and circadian neuronal activity in the DG. Given that the cAMP Responsive Element Protein binding (CREB) pathway exhibits rhythmicity in the hippocampus and considering CREB’s role in neuronal activity (Countryman et al., 2005), this underscores promising avenues for further research exploring the molecular link between aberrant light exposure and cognitive function.

Previous studies revealed the effects of altered light environments on emotional response associated with changes primarily in the hypothalamus and melatonin release (Fonken et al., 2009; An et al., 2020), but not in regions receiving direct light input from the ipRGCs such as the MeA (Hattar et al., 2006). Here, we show for the first time that altered light environments during adolescence impaired avoidance behaviors and disrupted clock gene expression and neuronal activity rhythm in the MeA. In the MeA, GABAergic neurons regulate avoidance and social behavior (Hong et al., 2014), of which 40% express SST neuropeptide (Keshavarzi et al., 2014). Among the heterogeneous MeA neuronal population, our studies revealed that a single light pulse at night selectively altered SST-expressing GABAergic subpopulation. In addition, we showed that exposure to LCD altered molecular clock and neuronal activity rhythms in SST neurons. Growing evidence suggests that circadian expression of SST in the amygdala plays a key role in avoidance behavior and in modulation of anxiety (Albrecht et al., 2013; Ahrens et al., 2018; Sun et al., 2020) and changes in SST neuronal density in the human amygdaloid complex has been associated with the pathogenesis of various neurological and psychiatric disorders (Pantazopoulos et al., 2017). Our analysis did not reveal any effect of LCD on daily SST expression patterns at the protein or mRNA level. However, we found profound changes in SST neuronal activity rhythms following LCD exposure, suggesting that LCD induced avoidance behavior by disrupting SST molecular clock and neuronal physiology in the MeA. Notably, the regulation of avoidance behaviors by light is absent in mice where ipRGCs are ablated (LeGates et al., 2012, 2014), indicating that ipRGCs are the primary sensory channel driving these behavioral responses. The MeA receives direct projection from ipRGCs and strong afferent from the accessory olfactory system (Raam and Hong, 2021), establishing it as a crucial region gating essential environmental cues to modulate avoidance/approach behaviors. Interestingly, lesions of the MeA alter light-enhanced startle and open-field behavior, along with other anxiety- and stress-related behavioral responses (Vinkers et al., 2010). Altogether, we suggest that the MeA might serve as a central hub controlling avoidance behavior in response to altered light environments. Future studies should reveal the role of SST neurons in the MeA in regulating avoidance behaviors after acute or chronic exposure to altered light environments.

Most of the major advances in our understanding of non-visual responses to light over the last two decades has been based upon studies in the mouse. The essential characteristics of light responses are conserved between nocturnal rodents and humans (Challet, 2007; Fonken et al., 2009; Fonken and Nelson, 2014). For example, light stimuli at night are known to trigger neuronal activation in the brain of both diurnal and nocturnal species, altering biological rhythms and behaviors (Brainard et al., 1985; Kornhauser et al., 1990; Colwell and Foster, 1992; Mahoney et al., 2001; Schumann et al., 2006; Challet, 2007). Importantly, recent studies found that blue wavelength nighttime light exposure induces alertness in both humans and nocturnal rodents (Vandewalle et al., 2010; Bourgin and Hubbard, 2016; Cajochen and Chellappa, 2016; Pilorz et al., 2016). These findings reconcile nocturnal and diurnal species through a common alerting response to blue light and indicate that artificial bright light can drive changes in mouse behavioral states analogous to the increase in alertness and arousal experienced by humans. In our paradigm we implemented LED light which has enhanced blue wavelengths (400–550 nm). Our analysis of voluntary wheel-running activity shows no effect of LCD exposure on circadian locomotor activity. Therefore, we suggest that data obtained in this work can be translationally relevant for understanding the effect of aberrant LED light exposure in humans.

However, it’s important to carefully consider the limitations of using rodent models related to light-induced behavioral responses. One of the strains used in our study, the C57BL/6 mouse, lacks detectable melatonin rhythms, unlike the CD1 mouse who have a light-sensitive melatonin secretion. Whether and how melatonin fluctuations contribute to altered affective responses associated with nighttime light exposure remains uncertain. Of note, melatonin receptors are absent in the amygdala in both humans and rodents (Ekmekcioglu, 2006), suggesting that the changes observed in the MeA might not be melatonin-dependent. Additionally, light acts as an aversive signal to rodents, which adds another layer of emotional significance in studies like ours. To gain a precise understanding of how nighttime light affects human emotions, future research needs to determine whether the pathways and mechanisms identified in rodents also apply to humans.

The escalating levels of nighttime illumination in the modern industrial world underscore the urgency of unraveling how neuronal circuits adapt to changes in the light environment. Emerging evidence indicates an association between altered light environments and neurophysiological and behavioral changes, with implications on mental health (Paksarian et al., 2020; Tancredi et al., 2022). To our knowledge, the study we present here is the first to characterize the effects of chronic light cycle disruption during pubertal development on brain regions regulating circadian rhythms, memory, and emotional responses. Our research provides new evidence highlighting the potential consequences of disrupted light environments during a critical period like adolescence on neuronal physiology and behaviors. Our findings elucidated the impact of altered light environments on memory and avoidance responses while shedding light on the molecular and cellular effects on MeA and hippocampal neuronal physiology. Nevertheless, additional research is necessary to ascertain whether light disruption induces enduring changes that persist into adulthood, potentially impacting the entirety of an individual’s lifespan. Given the ubiquitous nighttime over-illumination in contemporary society, our research highlights the adverse health implications it may entail for human health and emphasizes the importance of maintaining a consistent light environment for proper brain function during adolescence.

## Data availability statement

The data presented in the study are deposited in the Mendeley repository doi: 10.17632/s4gxwzhmfk.1.

## Ethics statement

The animal study was approved by the Institutional Animal Care and Use Committee (IACUC). The study was conducted in accordance with the local legislation and institutional requirements.

## Author contributions

PB: Conceptualization, Data curation, Formal analysis, Investigation, Methodology, Writing – original draft, Writing – review & editing. AS: Data curation, Formal analysis, Investigation, Writing – review & editing. YN: Data curation, Formal analysis, Investigation, Writing – original draft. AP: Conceptualization, Data curation, Formal analysis, Funding acquisition, Investigation, Methodology, Project administration, Resources, Supervision, Writing – original draft, Writing – review & editing.

## Supplementary material

Supplementary Figure S1 Cosinor analysis of Clock, Per1 and c-FOS expression in the SCN **(A-C)**, DG **(D-F)**, MeA **(G-I)** and SST neurons in the MeA **(J-L)**. Sine wave fits using linear harmonic regression assumed a 24h period for both control and LCD mice. Lines are superimposed on group means ± SEM (Control n=2 females and n=2 males; LCD n=2 females, n=2 males) for each ZT. R2 was used as a proxy of goodness-of-fit and p-values are shown when available; P<0.05 is considered significant. NaN represents an undefined value due to an undetectable rhythm.
