# Correction: Effects of chronic light cycle disruption during adolescence on circadian clock, neuronal activity rhythms, and behavior in mice

**DOI:** 10.3389/fnins.2025.1664442

**Published:** 2025-09-08

**Authors:** Pablo Bonilla, Alexandria Shanks, Yatin Nerella, Alessandra Porcu

**Affiliations:** Department of Drug Discovery and Biomedical Science, University of South Carolina, Columbia, SC, United States

**Keywords:** light cycle disruption, suprachiasmatic nucleus, dentate gyrus, medial amygdala, somatostatin, clock genes, neuronal activity, avoidance behavior

There was a mistake in Figure 3C as published. The panel labeled as Control-ZT22 was inadvertently duplicated from the LCD-ZT2 image. The panel labeled as Control-ZT16 was incorrectly selected and actually corresponds to Control-ZT22. This mistake occurred during figure assembly due to confusion while selecting representative figures from our blinded dataset. All data analyses and quantifications were conducted using the correct images, and we have verified that the conclusions of Per1 analysis remain valid and unaffected. The corrected Figure 3 appears below.

**Figure 3:**
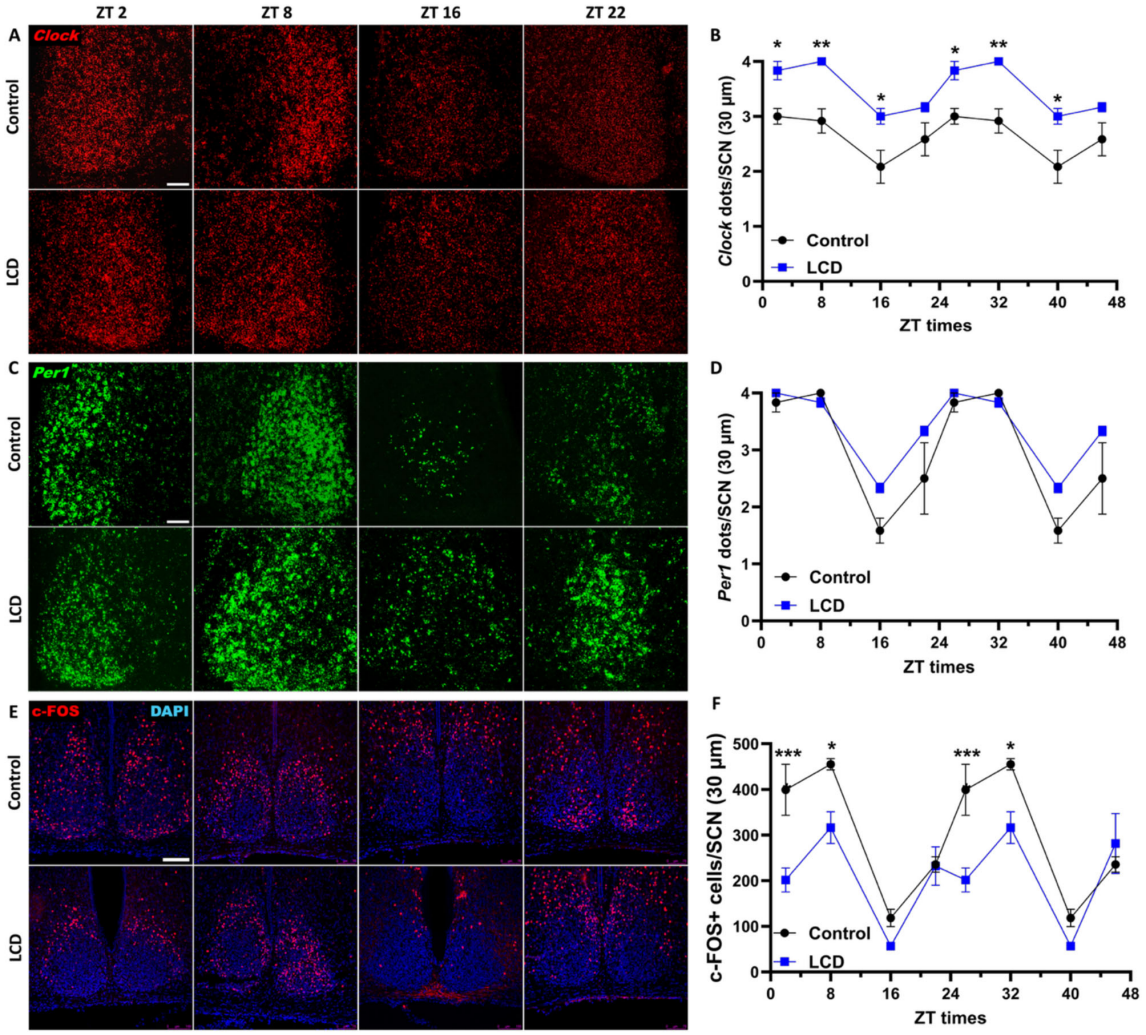
Daily expression of Per1, Clock and cFOS in the SCN. Representative confocal micrographs showing **(A)** *Clock* (red) and **(C)** *Per1* (green) mRNA expression detected by RNAscope and **(E)** c-FOS (red) detected by immunofluorescence at ZT2, ZT8, ZT16 and ZT22 in control and LCD mice (Scale bar 50 μm). Line graphs show **(B)** *Clock* mRNA expression (F_1, 32_ = 76.41, *p* < 0.0001 by two-way ANOVA with Šídák's multiple comparison posttest) and **(D)** *Per1* mRNA expression [*F*_(1, 32)_ = 10.17, *p* < 0.0032 by two-way ANOVA with Šídák's multiple comparison posttest] determined by semiquantitative scoring of Clock and Per1 dots and clusters per neuron, and **(F)** number of c-FOS positive neurons in the SCN [*F*_(1, 32)_ = 38.01, *p* < 0.0001 by two-way ANOVA with Šídák's multiple comparison posttest]. Data are shown as mean ± SEM. (Control *n* = 2 females and *n* = 2 males; LCD *n* = 2 females, *n* = 2 males) for the SCN in a 30-μm section; *p < 0.05, **p < 0.01, ***p < 0.001, ****p < 0.0001, two-way ANOVA (*post-hoc* test conducted with Šídák's multiple comparison test).

The original version of this article has been updated.

## Generative AI statement

Any alternative text (alt text) provided alongside figures in this article has been generated by Frontiers with the support of artificial intelligence and reasonable efforts have been made to ensure accuracy, including review by the authors wherever possible. If you identify any issues, please contact us.

