# Supplementary figures and images for: Effects of chronic light cycle disruption during adolescence on circadian clock, neuronal activity rhythms, and behavior in mice
Source: Front Neurosci. 2024 Jun 17;18:1418694. doi: 10.3389/fnins.2024.1418694 (PMC11215055; doi:10.3389/fnins.2024.1418694)

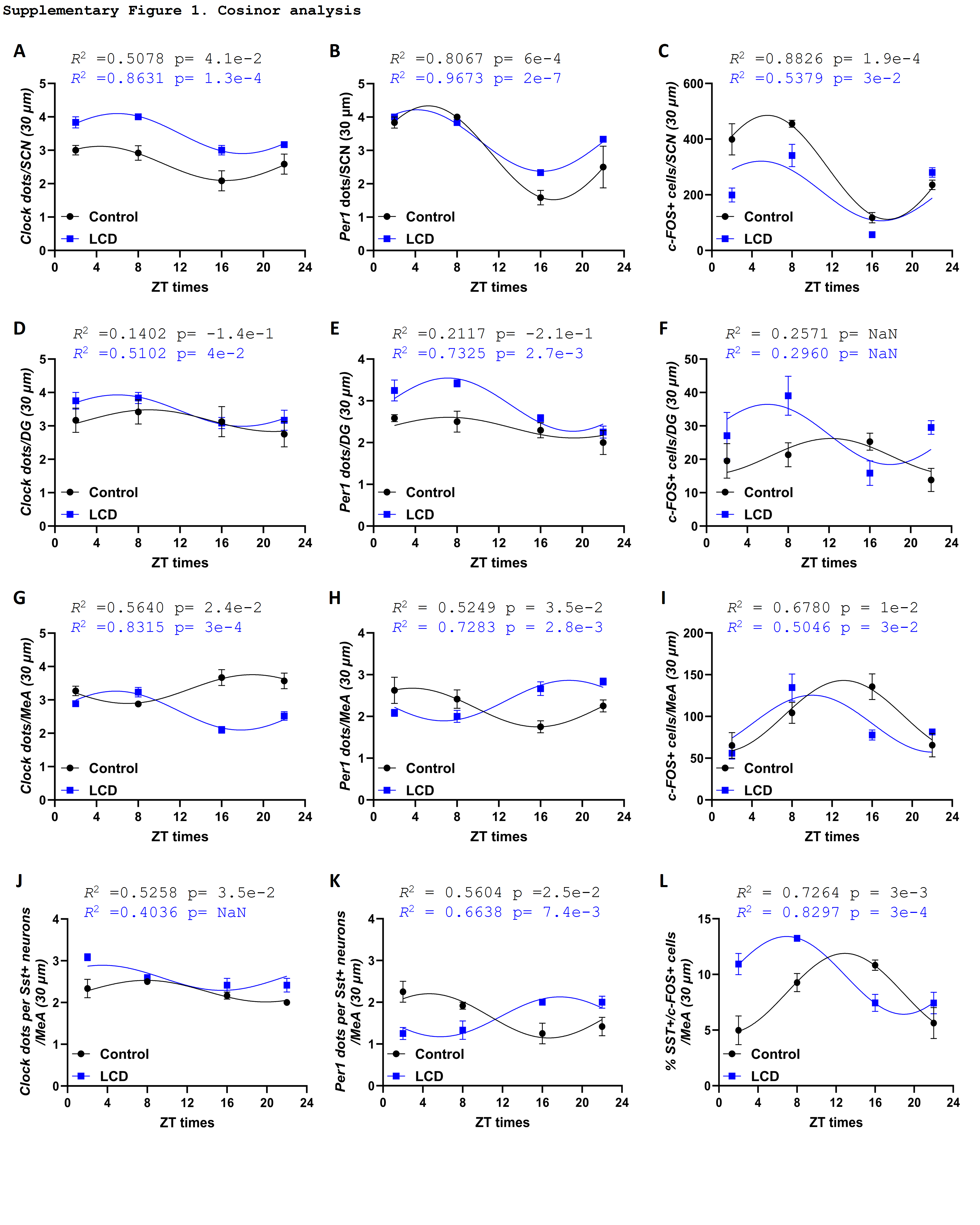

Supplement: Supplementary Figure S1 — Cosinor analysis of Clock, Per1 and c-FOS expression in the SCN (A-C), DG (D-F), MeA (G-I) and SST neurons in the MeA (J-L). Sine wave fits using linear harmonic regression assumed a 24h period for both control and LCD mice. Lines are superimposed on group means ± SEM (Control n=2 females and n=2 males; LCD n=2 females, n=2 males) for each ZT. R2 was used as a proxy of goodness-of-fit and p-values are shown when available; P<0.05 is considered significant. NaN represents an undefined value due to an undetectable rhythm. [file Image_1.TIF]
